# Symposium—Some Recent Advances in Hepatobiliary Disease and in the Clinical Aspects of Bile Acids

**Published:** 1990-06

**Authors:** 


### Symposium

### Some Recent Advances in Hepatobiliary Disease and in the Clinical Aspects of Bile Acids

### Held at Bristol Royal Infirmary, 22 September 1989

### FALLING INTO PLACE—THE PATHOGENESIS OF CHOLESTEROL AND PIGMENT STONES

**Martin C. Carey**, Harvard Medical School, Brigham and Women's Hospital and Harvard Digestive Diseases Center, Boston, MA, U.S.A.

#### CLASSIFICATION

The time honored classification of gallstones, attributed to Morgagni (1682–1771), Professor of Anatomy in Padua, labels gallstones according to what appears to be their predominant constituent, that is cholesterol or bile pigment, or a mixture of these substances. In this connection appearances are misleading and likely to lead to faulty ideas in attributing both etiologic causes to the stones, and potential for succesful dissolution. It is now generally agreed that all stones in gallbladders harboring multiple stones are of approximately the same composition. Further, if one plots percent of patients undergoing elective cholecystectomy for biliary pain, but without complications (i.e. no infection) versus per cent cholesterol in a single stone obtained from each gallbladder, one obtains a bimodal frequency distribution nomogram. In Western countries approximately 25% of gallstone patients have stones with <30% cholesterol by weight—most with <10% cholesterol. These are pigment gallstones and are composed of amorphous calcium bilirubinates that have undergone polymerization and oxidation, calcium carbonate and less frequenly calcium phosphate. Provided the bile is sterile and the stones contain no evidence of previous bacterial infection of bile (e.g. absence of bacterial rhamnose), no stones contain a percentage of cholesterol between 30 and 50% by weight. The remainder of the gallstone patients ~75%, possess stones with more than 50% cholesterol by weight. Further, the percent of patients in each decile, increases stepwise reaching a maximum for stones containing 90–100% cholesterol. Because anerobic bacterial infection of bile leads to calcium soap, calcium bile salt, calcium bilirubinate and even cholesterol precipitation, stones obtained from infectious bile, will have a continuum in percent cholesterol and hence are not classifiable on the basis of the aforegoing criteria. These stones are most often primary ductal in origin and are 'trivially' classified as 'brown' pigment stones. In contrast, the pigment stones formed in sterile gallbladder bile are 'black'. While brown 'stones' invariably contain precipitated calcium bilirubinate, the major components of 'brown' stones may be mucin glycoproteins, dead bacteria, calcium soaps, other calcium salts, deconjugated bile acids etc. These infectious stones will not be further discussed here, rather my focus will be upon the metabolic and physical-chemical origins of the two categories of gallbladder stones.

#### PHYSICAL CHEMISTRY

Common to all gallbladder stones either cholesterol or pigment is supersaturation, an essential but not sufficient physical-chemical defect. Because cholesterol (Ch) and monoanionic unconjugated bilirubinate ($HUCB^-$) are only sparingly soluble in water, they are transported in bile by binding to the major biliary lipids which are bile salts and diacylphosphatidylcholines (lecithins). Bile salt are present as monomers (equivalent to the critical micellar concentration), simple micelles (polymolecular aggregates *without* lecithin) and mixed micelles (polymolecular aggregates *with* lecithin); whereas lecithin which is otherwise insoluble in water, is solubilized as mixed micelles (with bile salts) and dispersed as unilamellar vesicles (closed bilayered membrane structures) containing little or no bile salts. Bile salt monomers, as well as the lipid aggregates can bind both Ch and $HUCB^-$ and act as their 'carriers' in bile. Hence supersaturation can occur if there is an excess of biliary Ch or $HUCB^-$ or if there is a deficiency in the 'carriers'. From secretory studies, we now know that all Ch gallstone patients have either hypersecretion of hepatic Ch or hyposecretion of bile salts into bile. Rarely a combination of both secretory defects may occur. Once bile salt monomer plus simple and mixed micellar solubility of Ch is exceeded, lecithin vesicles appear in bile and disperse, albeit in an unstable way, the excess Ch molecules as lecithin/Ch vesicles. If these become unstable as they do in Ch gallstone patients they aggregate and fuse, grow in size and after a couple of days they 'spawn' Ch monohydrate crystals. Finally the high Ch content of vesicles leads to continued growth of the microcrystals to produce crystals of sufficient size to agglomerate into macroscopic gallstones.

While a key physical chemical characteristic of Ch stone biles is the presence of vesicles carrying excess Ch, pigment stone biles, in contrast, are uniformly devoid of lecithin/Ch vesicles. This immediately suggests that pigment stone gallbladder biles are missing an important carrier not only for binding $HUCB^-$ but also for binding ionic calcium ($Ca^{2+}$). The absence of these carriers may be due to hypersecretion of bile salts (which appears unlikely) or hyposecretion of hepatic Ch. While secretory and production studies of $HUCB^-$ in human biles are lacking, there appears to be an absolute increase in biliary UCB (which forms $HUCB^-$ at peri-neutral biliary pH values), either from hypersecretion or *de novo* formation from bilirubinate conjugates, the primary form in which bilirubin is secreted into bile. Since vesicles are absent in such biles, the nucleation sequence described for Ch, cannot take place but rather, precipitation of $Ca(HUCB^-)_2$ must occur from HUCB− supersaturated bile salt monomers and from simple and mixed micelles.

#### PATHOGENESIS

Formation of either Ch or pigment stones in the gallbladder are not single diseases but the complications of many different diseases that have stones as their common physical-chemical endpoints.

#### CHOLESTEROL GALLSTONES

A. Multiple etiologic factors in hypersecretism of biliary Ch are

(i) increased Ch input to the liver from increased hepatic uptake of low density lipoproteins (LDL), high density lipoproteins (HDL) and chylomicron remnants such as in estrogen- and dietary-induced gallstones.

(ii) Increased Ch synthesis in the liver (and extrahepatic tissues) as in gallstones secondary to human obesity and hypertriglyceridemic states.

(iii) Decreased Ch catabolism in the liver either to form bile salts *de novo* or cholesteryl esters for storage, nascent HDL and very low density lipoprotein (VLDL) production (as in progestogenic and 'fibric acid' induced gallstones).

B. Multiple etiologic factors in hyposecretion of bile salts are

(i) decreased *de novo* bile salt synthesis such as in Ch gallstones caused by human aging, and congenital and acquired bile salt synthetic defects.

(ii) decreased enterohepatic cycling of bile salts from motor, hormonal or receptor defects ("idiopathic"; these defects have not been defined at the molecular level).

(iii) Torrential gastrointestinal loss of bile salts uncompensated by *de novo* hepatic synthesis. Although this disorder has been claimed as responsible for Ch gallstone formation in chronic ileal disease (Crohn's), ileal resection, bypass, or acidic small bowel states (Cystic Fibrosis), it now appears that pigment gallstones are the final end-result of these diseases.

### PIGMENT GALLSTONES

C. Multiple etiologic factors in hypersecretion or formation of excess $HUCB^-$. These factors are poorly defined but the following are postulated:

(i) Increased biliary secretion of UCB in chronic hemolytic states.

(ii) Increased secretion of photoisomerized UCB.

(iii) Increased activity of $\beta$-glucuronidase of biliary tree or hepatic origin.

(iv) Decreased activity of biliary $\beta$-glucaro-1-4-lactone, an inhibitor of $\beta$-glucuronidase.

(v) Decreased binding of UCB to lipids in bile due to the absence of vesicles i.e. absence of Ch supersaturation.

D. Multiple etiologic factors in hypersecretion and/or increased activity of biliary calcium.

(i) Any chronic hypercalcemic states, (e.g. hyperparathyroidism, sarcoidosis, immobilization etc.).

(ii) Diminished binding to/or absent vesicles, especially from hyposecretion of biliary Ch (e.g. alcoholic cirrhosis)

(iii) Decreased binding to micelles from hyposecretion of bile salts.

### MODIFIERS OF NUCLEATION

Modifiers are kinetic and motor defects that are essential for the thermodynamic defect of supersaturation to result in gallstones. These defects are best defined for Ch crystal nucleation and gallstone formation. Present in normal bile are anti-nucleating glycoproteins as well as possibly other substances, some of which prevent vesicle fusion and aggregation and nucleation, whereas others appear to be anti-crystal growth substances. It is currently thought that these potent inhibitors are absorbed to vesicles or to the active growth sites on the Ch-crystal surfaces where they block further crystal growth and perhaps suppress aggregation. In Ch stone biles, there appears to be both excess of mucin glycoproteins as well as soluble glycoproteins that act as powerful accelerators of vesicle fusion, growth and crystal nucleation. Initially Ch crystals, within the gallbladder, are minute and possibly too small to be trapped in the viscus, unless other modifiers are present concurrently. This appears to be the case. In Ch gallstone patients there is primary gallbladder hypomotility of uncertain etiology. Hypomotility may be a primary or secondary smooth muscle defect, occurring in response to some intravesicle chemical "trigger" that is absorbed by the mucosa. In some patients hypomotility may be related to defective cholecystokinin release from the upper small intestine or the gallbladder's response to cholecystokinin. The result is a decreased and delayed gallbladder ejection fraction and increased residual volume.

A further list of factors promoting retention of minute Ch crystals and subsequent gallstone formation are (i) Ch monohydrate is more dense than bile; the Ch crystals sink to the fundus of the gallbladder and cannot reach the cystic duct since this outlet is at the top of the viscus when the subject is upright. (ii) Mucin gel presumably aids in the retention of crystals at the gallbladder's fundus since mucin also depends upon normal gallbladder contraction for its elimination (iii) Mucin gel may also act as intercrystalline cement facilitating agglomeration of small Ch microcrystals. (iv) The site of nucleation is most likely not in the fluid intravesicle 'phase' of bile but juxtamucosal in the visco-elastic mucin gel on the gallbladder wall. It is here that mucin gel is first formed and where Ch-supersaturated vesicles and micelles of bile are concentrated by solvent drag secondary to dehydration of bile. Hence the organic mucin matrix, gallbladder hypomotility, as well as other uncharacterized substances my greatly modify and facilitate the chances of Ch crystal formation and retention within the gallbladder.Whether kinetic and motor defects modify calcium bilirubinate formation and precipitation within the gallbladder are unknown but they remain a real possibility. Clearly, the more detailed our knowledge of all the factors involved in the progression of abnormal bile to the stone 'end-point' the more developed our understanding of the pathogenesis of stones will become. This is particularly important for developing effective programs for the prevention of both cholesterol and pigment stone formation in human beings.

### FURTHER READING

1. CAREY, M. C. and CAHALANE, M. J. (1988) Whither Biliary Sludge? (Review) *Gastroenterology* **95**, 508–523.
2. CAHALANE, M. J., NEUBRAND, M. W. and CAREY, M. C. (1988) Physical-chemical pathogenesis of pigment gallstones. *Sem Liver Dis* **8**, 317–328.
3. CAREY, M. C. (1989) Formation of cholesterol gallstones: The New Paradigms, in Trends in Bile Acid Research. (Paumgartner, G., Stiehl, A., Gerok, W., eds.) Dordrecht, The Netherlands, Kluwer Academic, 259–281.
4. CAREY, M. C. (1990) Overview of the pathogenetic events in biliary stone formation. In Proc. Holland Dis Dis Week, (Tytgat, G. N. J., ed.), Stuttgart, Georg Thieme. (in press).

### SYMPTOMATIC AND SILENT GALLSTONES IN THE COMMUNITY: NEW DATA FROM BRISTOL

**K. W. Heaton, F. E. M. Braddon, R. A. Mountford, A. O. Hughes, P. M. Emmett and S. Ghosh**, Departments of Medicine and Epidemiology, University of Bristol

This is a just-completed cross-sectional population survey involving a stratified random sample of 838 men aged 40–69 and 1058 women aged 25 (72.2% of those approached). Ultrasonography detected 92 cases of gallstones and a further 51 subjects had already undergone cholecystectomy.

All subjects were questioned about their habitual intake of sugar- and fibre-containing foods and fasting plasma was analysed for insulin, glucose, total cholesterol, triglycerides, $HDL_2$ and $HDL_3$ cholesterol. Information was also obtained on parity (including abortions), weight change since age 20, waist and hip circumferences, bowel habit, and usual stool form on a validated scale from 1 (scybala) to 7 (watery).

In a parallel case-control study, 80 subjects with unsuspected gallstones are being compared with age- and sex-matched stone-free controls with respect to whole-gut transit time and nutrient intakes (assessed from a 4-day weighed record).

Analysis of these extensive data is proceeding. By mid-September 1989 the following facts had emerged:

(1) Gallstone prevalence flattens off in middle-aged women in Bristol; otherwise prevalence rises with age in both sexes, reaching 22.4% in women aged 60–69;

(2) the incidence of biliary pain was very low in people with gallstones and even lower (<5%) if the standardised questionnaire was supplemented by normal history-taking;

(3) women are more likely than men to have symptoms from their gallstones and to undergo cholecystectomy.

### THE BILIARY TRACT IN PATIENTS WITH GALLSTONE PANCREATITIS

**C. P. Armstrong**, Dept of Surgery, Bristol Royal Infirmary

The concept of gallstone migration is now generally accepted although the reason why only a few patients with gallstones develop pancreatitis is unknown.

One thousand five hundred patients undergoing surgery for gallstones were prospectively studied; 203 (13.5%) had a history of gallstone pancreatitis. Patients with pancreatitis had significantly more gall bladder stones 22.3±14.1 versus 15.2±11.6, P<0.02, and 74% had more than 10 stones in contrast to 43% control patients (P<0.001). The smallest gall bladder stone was considerably smaller in the pancreatitis patients; 2.4±1.6 mm versus 5.9±4.1, P<0.001; and 93% had stones of 3 mm or less contrary to 37% of controls (P<0.001). The cystic duct was wider in pancreatitis patients; 4.5±2.0 mm versus 3.3±1.9 mm, P<0.001; and 58% had a cystic duct of 4 mm or larger compared with 17 per cent of controls (P<0.001). Stone passage through the cystic duct was possible in all patients with pancreatitis but in only half the controls. The common bile duct diameter in patients with pancreatitis was independent of stones within its lumen. Choledochal calculi were of equal size in the two groups. Pancreatic-duct reflux was far more commonly observed on the operative cholangiograms of pancreatitis patients; 67.0 versus 16.6%, P<0.0001.

These observations clearly affirm the importance of small stone migration in the pathogenesis of acute pancreatitis. Patients with gallstones who develop pancreatitis have a biliary tract that predisposes to gallstone migration and pancreatic-duct reflux.

### URSODEOXYCHOLIC ACID: A NEW WONDER DRUG FOR HEPATO-BILIARY DISEASE?

**Malcolm C. Bateson**, Department of Gastroenterology, General Hospital, Bishop Auckland, County Durham

Ursodeoxycholic acid is normally a minor component of human bile. Treatment with ursodeoxycholic acid leads to enrichment of bile so that up to 50% of the bile acids become ursodeoxycholic acid. This reduces biliary cholesterol levels, and cholesterol gallstone formation can be prevented in risk populations. Existing gallstones can be dissolved. This is only very effective for small stones, so the additional use of extra-corporeal shock wave lithotripsy to reduce the size of larger stones is an attractive option. The combined use of cheno-deoxycholic acid with ursodeoxycholic acid may be even more effective therapy for gallstones, leading to more rapid and complete dissolution, and possibly reducing the risk of induced gallstone calcification which is the only definite side-effect of ursodeoxycholic acid therapy.

It was found by chance that gallstone patients with co-existing liver disease derived additional benefit from bile acid therapy. This is best established in primary biliary cirrhosis. Treatment with ursodeoxycholic acid 750 mg. daily improves symptoms like pruritus and diarrhoea. Abnormally elevated serum enzyme levels are reduced after a few weeks, and this fall continues until four to six months, the effects being sustained thereafter. No improvement in steatorrhoea was shown by triolein breath tests performed before and after four months' therapy, and the possibility of a deterioration could not be excluded. Liver histology did not usually change after four months, but there was sometimes a reduction in inflammatory cell infiltrate. The effect on survival is not known, and it will take many years to assess. There is no definite advantage in using larger doses of ursodeoxycholic acid than 750 mg. daily, but smaller doses may be just as effective. Though evidence is patchy and incomplete, benefit from ursodeoxycholic acid has also been reported in chronic active hepatitis, primary sclerosing cholangitis and biliary atresia, as well as in bile reflux gastritis and non-specific dyspepsia.

The only cure for primary biliary cirrhosis is liver transplantation. Colchicine and azathioprine may prolong life but results are equivocal. Attempts to control liver inflammation have usually involved toxic drugs like prednisolone, penicillamine, cyclosporin, methotrexate and nalmefene. Since ursodeoxycholic acid controls symptoms and appears to be harmless, it has a claim to be the treatment of first choice in primary biliary cirrhosis if any drug therapy is required.

### REFLUX OESOPHAGITIS: DO BILE ACIDS HAVE A ROLE?

**D. C. Gotley, A. P. Morgan and M. J. Cooper**
University Department of Surgery, Bristol Royal Infirmary

Reflux oesophagitis is attributed to the digestive action of acid and pepsin on the oesophageal mucosa. Bile acids are also thought to play a role (and may be responsible for some treatment failures), but have not been studied in the oesophagus.

Fifty-two patients with gastro-oesophageal reflux, none of whom had previous gastric surgery, were studied by simultaneous oesophageal aspiration and pH monitoring between 1700–0900 hr. Aspirates, in 2 hourly aliquots, were assayed for conjugated and unconjugated bile acids and pepsin. The results were compared according to the degree of oesophagitis determined by endoscopy. Patients with oesophagitis had greater acid reflux than refluxers with a normal oesophagus (18 vs 8.3 for % time pH<4; P<0.05). Conjugated bile acids were found in aspirates from 75% of patients, and were equally distributed among the different grades of oesophagitis. Only 2% of 364 aspirates contained concentrations known to be cytopathic to oesophageal mucosa (>1 mmol/l), and no bile acids were unconjugated. Patients with complicated oesophagitis (stricture, Barrett's oesophagus) had higher pepsin concentrations than those with uncomplicated erosive oesophagitis (153 vs 46 ug/ml P<0.001).

The results affirm the importance of acid and pepsin in the pathogenesis of reflux oesophagitis, and suggest that bile acids do not play a major role in this disease.

### LARGE BOWEL CANCER: THE ROLE OF SECONDARY BILE ACIDS

**R. W. Owens BSc PhD**, PHLS, CAMR., Division of Biotechnology, Porton Down, Salisbury, Wilts., SP4 0JG

The notion that bile acids may be important in the aetiology of colorectal cancer (CRC) was forwarded by Hill *et al.* in

1971(1). In a population study faecal bile acid (FBA) concentration was shown to correlate positively with the incidence of CRC and later studies (2,3) showed a similar correlation in CRC case versus control studies. In brief, a hypothesis was presented and tested during the following decade based on the following dictat: "CRC may be caused by the production of carcinogenic compounds (unsaturated bile acids) from the metabolism of bile acids by intestinal bacteria within the large bowel". Although bacteria (especially *Clostridia*) were isolated and identified from intestinal contents capable of elaborating the necessary enzymes, the products, after exhaustive testing, were not found to be potent mutagens or carcinogens (4). However, in many "cancer" models it has been shown that the secondary bile acid metabolites principally lithocholic acid (LCA) and deoxycholic acid (DCA) exhibit both co-mutagenic and co-carcinogenic properties. It has been concluded, therefore, that the positive correlations observed in population studies between FBA concentration and CRC incidence is probably due to the cancer promoting properties of these lipids.

With the advent of more sophisticated methods [Owen *et al.*, 1984(5)] for lipid analyses, recent studies have shown that in CRC case control studies the faecal bile acid profile is more important than FBA concentration especially as a marker of CRC (6).

In summary, the LCA:DCA ratio in CRC patients ($1.81 \pm 0.19$) is diametrically opposed to that of control subjects ($0.87 \pm 0.07$) such that 72% of CRC patients have an abnormal ratio (>1:0) compared to only 25% of controls. Of interest is that the LCA:DCA ratio correlates positively with adenoma size and severity of CRC.

It is concluded that the LCA:DCA ratio may be a useful adjunct to future screening procedures for CRC.

### REFERENCES

1. HILL, M. J., DRASAR, B. S., ARIES, V. C., CROWTHER, J. S., HAWKESWORTH, G. M. and WILLIAMS, R. E. O. (1971). Bacteria and the Aetiology of Large Bowel Cancer. *Lancet* **i**, 95–100.
2. HILL, M. J., DRASAR, B. S., WILLIAMS, R. E. O., MEADE, T., SIMPSON, J. and MORSON, B. C. (1975). Faecal Bile Acids, Clostridia and the Aetiology of Cancer of the Large Bowel. *Lancet* **i**, 535–539.
3. REDDY, B. S. and WYNDER, E. L. (1977). Metabolic Epidemiology of Colon Cancer. Faecal Bile Acids and Neutral Sterols in Colon Cancer Patients and Patients with Adenomatous Polyps. *Cancer* **39**, 2533–2539.
4. McKILLOP, C. A., OWEN, R. W., BILTON, R. F. and HASLAM, E. A. (1983). Mutagenicity Testing of Steroids Obtained from Bile Acids and Cholesterol. *Carcinogenesis* **4**, 1179–1183.
5. OWEN, R. W., THOMPSON, M. H. and HILL, M. J. (1984). Analysis of Metabolic Profiles of Steroids in Faeces of Healthy Subjects Undergoing Chenodeoxycholic Acid Treatment by Liquid-Gel Chromatography and Gas-Liquid Chromatography—Mass Spectrometry. *J. Steroid Biochem.* **21**, 593–600.
6. OWEN, R. W., DODO, M., THOMPSON, M. H., and HILL, M. J. (1987). Faecal Steroids and Colorectal Cancer. *Nutr. Cancer* **9**, 73–80.

